# Comparison of Epidermal Growth Factor Receptor Mutations between Metastatic Lymph Node Diagnosed by EBUS-TBNA and Primary Tumor in Non-Small Cell Lung Cancer

**DOI:** 10.1371/journal.pone.0163652

**Published:** 2016-09-29

**Authors:** Hyo Jae Kang, Bin Hwangbo, Jin Soo Lee, Moon Soo Kim, Jong Mog Lee, Geon-Kook Lee

**Affiliations:** 1 Center for Lung Cancer, Research Institute and Hospital, National Cancer Center, Goyang, Korea; 2 Department of Pathology, Research Institute and Hospital, National Cancer Center, Goyang, Korea; Baylor College of Medicine, UNITED STATES

## Abstract

**Introduction:**

Although the use of endobronchial ultrasound-guided transbronchial needle aspiration (EBUS-TBNA) is increasing for epidermal growth factor receptor (EGFR) testing in lung cancer, the discordance rate in EGFR mutations between lymph node (LN) samples obtained by EBUS-TBNA and primary tumor (PT) is not well known. Thus, we compared the EGFR mutation status of LN samples obtained by EBUS-TBNA and PTs to estimate the efficacy of using EBUS-TBNA specimens for EGFR testing in advanced, non-squamous, non-small cell lung cancer (NSCLC).

**Materials and Methods:**

Using data of patients from the EBUS-TBNA database (N = 1914) obtained between January 2009 and January 2013, we identified 100 treatment-naïve, advanced, non-squamous NSCLC patients (stage 3 and 4) with matched LN specimens obtained by EBUS-TBNA and PT specimens. Of these, 74 patients with paired specimens were feasible for EGFR mutation analysis, which we performed using a direct sequencing method.

**Results:**

Of the 74 cases, at least one major [exon 19 deleted (19del) and L858R] or minor (T790M, exon 20 insertion, and other point mutations) EGFR mutation was detected in 31 cases (41.9%), which included PT (n = 31, 41.9%) and LN (n = 28, 37.8%) specimens. Major mutations were detected in 25 PT (33.8%, 19del = 13, L858R = 12) and 22 LN (29.8%, 19del = 11, L858R = 11) specimens. The discordance rate in major mutations between matched PT and LN specimens was 4.1% (3/74). Among minor mutations, T790M was detected in LN specimen only in 2 cases with L858R in PT and LN. The discordance rate major and minor EGFR mutations combined between matched PT and LN specimens was 12% (9/74).

**Conclusions:**

We observed a high concordance rate of major EGFR mutations between matched LN specimens sampled by EBUS-TBNA and PTs, suggesting that LN samples obtained by EBUS-TBNA from advanced non-squamous NSCLC patients are effective for use in EGFR mutation testing.

## Introduction

The detection of epidermal growth factor receptor (EGFR) tyrosine kinase inhibitor (TKI)-sensitizing mutations is important in guiding the treatment of advanced non-small cell lung cancer (NSCLC). Clinical trials have confirmed that the response rate to the TKIs gefitinib and erlotinib in patients with EGFR mutations is approximately 70–80% [1–3]. When considering first-line therapy options for patients with NSCLC, EGFR mutation testing is highly recommended to determine whether the patient should undergo EGFR-TKI treatment or chemotherapy [4].

Obtaining an adequate amount of tissue at the time of lung cancer diagnosis is essential for accurately diagnosing the histologic differentiation and molecular status of the tumor, which includes identifying EGFR mutations. For tissue acquisition of lung cancer, targeting the primary tumor (PT) is not mandatory, and metastatic lymph nodes (LNs) or other metastatic sites can be the first diagnostic target [5]. The ideal sampling site and method should allow for the acquisition of an adequate amount of sample in a least invasive manner.

Endobronchial ultrasound-guided transbronchial needle aspiration (EBUS-TBNA) is a minimally invasive method that shows high value in diagnosing mediastinal LNs. Currently, EBUS-TBNA is recommended over mediastinoscopy for initial mediastinal staging [6]. In addition, EBUS-TBNA offers high sensitivity for the diagnosis of lung cancer by targeting metastatic nodes or accessible parenchymal lesions. Importantly, EBUS-TBNA specimens can also be used for EGFR mutation testing. For instance, Navani et. al. reported the successful use of EBUS-TBNA specimens for EGFR mutation analysis in 90% of patients in whom mutation analysis requested [7]. The use of EBUS-TBNA as an initial method for tissue acquisition and EGFR testing in lung cancer patients is increasing.

However, there are concerns regarding the choice of tissue sampling site such as the difference caused by sampling techniques and the potential for differences in molecular status between the PT and metastatic sites. A number of studies have evaluated the difference in EGFR mutation status between the PTs and metastatic LNs. According to a meta-analysis that included data from nine publications, the overall discordant rate of major EGFR mutations, including exon 19 deletion and exon 21 L858R, was 12.2% (range 4.5–28.6%), with PT and LN mutation rates of 26.4% and 19.9%, respectively[8]. However, in most of those studies, the EGFR mutation status was tested using surgically resected PT and LN specimens from operable lung cancer patients [9–15]. Therefore, these studies failed to address whether EBUS-TBNA targeting metastatic LNs can be used effectively for EGFR testing in patients with advanced, inoperable lung cancer. Until now, only one study, using a small number of patients (n = 14), has compared EGFR mutations between LN samples obtained by EBUS-TBNA and surgically resected PTs, and they found a discordant rate in major EGFR mutations of 7.1%., with PT and LN mutation rates of 28.6% and 21.4%, respectively[16]. Therefore, in this study, we analyzed EGFR mutations using direct sequencing in matched LN samples obtained by EBUS-TBNA and PT, to estimate the efficacy of using EBUS-TBNA samples for EGFR mutation testing in advanced, non-squamous NSCLC.

## Methods

### Patients

We retrospectively reviewed the data obtained between January 2009 and January 2013 in the EBUS-TBNA database (N = 1914) in the National Cancer Center, Goyang, Korea. We selected 895 patients with non-squamous NSCLC. Of these, we enrolled 100, treatment-naïve, advanced, non-squamous NSCLC patients (stage 3 and 4) with matched PT specimens, who informed consented to the use of their tumor specimens for genetic molecular testing, in this study. We accepted PT samples obtained by various methods including surgery, if they were obtained before chemo- or radiation therapy, and if the interval between PT sampling and EBUS-TBNA was less than 2 months. This study is the NCCNCS-13765 trial performed at the National Cancer Center, Korea. The Ethical Review Committee (National Cancer Center Institutional Review Board) of our institution approved the protocol.

### EBUS-TBNA

We performed EBUS-TBNA using an ultrasonic bronchoscope with a linear scanning transducer (convex probe-EBUS, BF-UC260FOL8, Olympus, Tokyo, Japan) and a dedicated ultrasound processor (EU-C2000, Olympus). We performed needle aspiration using a dedicated 22-gauge needle (NA-201SX-4022, Olympus). All aspirate specimens were placed into a solution of 10% neutral-buffered formalin. The samples were centrifuged and processed to make formalin-fixed, paraffin-embedded (FFPE), cell blocks.

### EGFR mutation analysis with direct sequencing0

First, we checked for the adequacy of material in the FFPE blocks of EBUS-diagnosed LN and PT specimens of the enrolled patients by microscopy with hematoxylin and eosin staining on the FFPE tissue or cell-block sections (DR GK Lee). If the proportion of tumor cells was more than 50% and number of tumors cells was more than 200, we considered the sample feasible for EGFR testing. When the proportion of tumor cells was less than 50%, we still considered the sample feasible for EGFR testing if approximately 200 tumor cells were available following microdissection. If more than one PT or LN specimen was available for a patient, then a pathologist selected the most suitable one. Of the 100 PT-LN specimens, 78 PT and 89 LN samples were feasible for EGFR mutation analysis. Finally, 74 patients with paired specimens were feasible for EGFR analysis. Of the 148 specimens from the 74 patients, EGFR mutation testing with direct sequencing had been performed previously as clinical practice in 61 specimens (PT = 45, LN = 16). Thus, we performed EGFR direct sequencing with the other 87 specimens (PT = 29, LN = 58) in October of 2013 using the same direct sequencing method that was used on[17, 18].

Briefly, for each specimen, we used four microsections (10-μm thickness) of the FFPE tissues and extracted the genomic DNA using a QIAamp DNA Mini kit (Qiagen, Valencia, CA). We amplified the tyrosine kinase domain of the EGFR-coding sequence, which includes exons 18, 19, 20, and 21, and we purified and sequenced the PCR amplicons in an automatic ABI Prism 3100 Analyzer. We performed all sequencing reactions in both the forward and reverse directions, and we repeated all PCR direct sequencing reactions twice to confirm the results.

### Review of medical records

We reviewed the following baseline characteristics of the 74 patients who were tested for EGFR mutations: age, gender, smoking history, histologic types, tumor stage, LN location tested, size of PT and LN, maximum standardized uptake value (mSUV) of PT and LN from Positron Emission Tomography—Computed Tomography (PET/CT), number of aspirations by EBUS-TBNA, and PT tissue acquisition method. We also reviewed the use of selective EGFR-TKIs and the tumor response rates. Objective tumor responses were assessed at 3 months after initiation of TKI treatment. According to the RECIST criteria, a complete response (CR) means disappearance of all target lesions and a partial response (PR) is defined as at least a 30% decrease in the sum of the diameters of target lesions. Progressive disease (PD) is at least a 20% increase in the sum of the diameters of target lesions and stable disease (SD) means neither sufficient shrinkage to qualify for PR nor sufficient increase to qualify for PD.

### Statistical Analysis

We used chi-square (χ^2^) test or Fisher’s exact test for evaluating categorical data between groups. A p value of <0.05 was considered statistically significant. We performed all statistical analyses using Stata statistical software V.10.0 (Stata Corp, College Station, Texas, USA).

## Results

### Patient and specimen characteristics

Table 1 shows the baseline characteristics of the 74 patients whose samples were analyzed for EGFR mutations. Females comprised 40.5%, and never smokers were 44.6%, of the patients. Most patients had adenocarcinoma (n = 72, 97.3%). Of the 74 patients, 30 had stage 3 (40.5%), and 44 had stage 4 (59.5%), tumors. N2-3 or N1 LNs were tested in 67 (90.5%) and 7 (9.5%) of the patients, respectively. The mean size of PT and LNs were 43.4mm and 13.3 mm, respectively. The mean of mSUV of PT and LNs were 11.1 and 6.6, respectively in evaluable cases. The mean number of aspirations by EBUS-TBNA per LN was three. Of the PT specimens, 30 (40.5%) were obtained by bronchoscopic methods, including EBUS-TBNA and endobronchial biopsy, 30 (40.5%) were obtained by transthoracic needle biopsy, and 14 (18.9%) were obtained by surgery. The number of PT and LN specimens with a tumor proportion >50% was 54 (73.0%) and 52 (70.3%), respectively (p = 0.72), and the number of PT and LN specimens with a tumor proportion ≥20% was 62 (83.8%) and 63 (85.1%), respectively (p = 0.82).

**Table 1:** Baseline characteristics of 74 patients.

Characteristics		
Age, years, median (range)		63 (39–84)
Gender, n (%)	Male	44 (59.5)
	Female	30 (40.5)
Smoking status, n (%)	Never-smoker	33 (44.6)
	Former or current smoker	41 (55.4)
Lung cancer histology, n (%)	Adenocarcinoma	72 (97.3)
	Large cell neuroendocrine carcinoma	2 (2.7)
Lung cancer stage, n (%)	IIIA	24 (32.4)
	IIIB	6 (8.1)
	IV	44 (59.5)
Locations of LNs tested, n (%)	2R	13 (17.6)
	2L	1 (1.4)
	4R	25 (33.8)
	2R/4R	2 (2.7)
	4L	12 (16.2)
	7	14 (18.9)
	10R	2 (2.7)
	10L	1 (1.4)
	11R	1 (1.4)
	11L	3 (4.1)
Size of PT and LNs	Long diameter(mm) of PT, mean(SD)	43.4 (18.2)
	Short diameter(mm) of LN, mean(SD)	13.3(6.3)
mSUV in FDG-PET of PT and LNs	mSUV of PT, mean(SD), n = 57*	11.1(5.4)
	mSUV of LNs, mean(SD), n = 43*	6.6 (3.4)
Number of aspirations by EBUS-TBNA for LNs, Mean (range)		3 (2–5)
Tissue acquisition method for PT, n (%)	Surgery	14 (18.9)
	Endobronchial biopsy	10 (13.5)
	Transbronchial lung biopsy	3 (4.1)
	EBUS-TBNA	17 (23.0)
	Transthoracic needle biopsy (TTNB)	30 (40.6)

* Available number of cases

### EGFR mutations results of PT and LN specimens

The results of the EGFR mutation analyses are presented in Table 2. Among the 74 cases, 31 (41.9%) showed at least one EGFR mutation, including the PT and LN specimens. Major (exon 19 deletion and exon 21 L858R mutation) or minor EGFR mutations were found in 31 PT (41.9%) and 28 LN (37.8%) specimens. Major mutations were detected in 25 PT (33.8%, 19del = 13, L858R = 12) and in 22 LN (29.7%, 19del = 11, L858R = 11) specimens. In three cases, we detected major EGFR mutations in the PT specimens that were not detected in the matched LN specimens (19del in cases 12 and 13, L858R in case 25). Therefore, we determined a discordant rate in major mutations between matched PT and LN specimens of 4.1% (3/74). Of the patients with major mutations, the discordant rate was 12% (3/25). The details of these three cases with discrepancy in major mutations are presented in Table 3. In the three patients, the proportion of tumor cells in the LN specimens was 5–10%, and the number of aspirations by EBUS-TBNA for LN sampling was two or three.

**Table 2:** Results of 31 patients with EGFR mutation(s) and response to tyrosine kinase inhibitor.

Pt. #	Primary tumor		EBUS diagnosed LN		TKI, sequence	Response to TKI
	Exon	Mutation	Exon	Mutation		
1	19	Deletion (E746_A750del)	19	Deletion (E746_A750del)	Gefitinib, 3^rd^	PR
2	19	Deletion (L747_S751del)	19	Deletion (L747_S751del)	Gefitinib, 2^nd^	PR
3	19	Deletion (E746_P753del)	19	Deletion (E746_P753del)	None	
4	19	Deletion (E746_A750del)	19	Deletion (E746_A750del)	Gefitinib, 1^st^	SD
5	19	Deletion (E746_S752del)	19	Deletion (E746_S752del)	Gefitinib, 1^st^	PR
6	19	Deletion (E746_A750del)	19	Deletion (E746_A750del)	Gefitinib, 1^st^	PR
7	19	Deletion (L747_A750del)	19	Deletion (L747_A750del)	Erlotinib, 2^nd^	PR
8	19	Deletion (L747_T751del)	19	Deletion (L747_T751del)	Erlotinib, 2^nd^	PR
9	19	Deletion (E746_A750del)	19	Deletion (E746_A750del)	None	
10	19	Deletion (L747_S752del)	19	Deletion (L747_S752del)	Erlotinib, 1^st^	PR
11	19	Deletion(L747_S751del)	19	Deletion (L747_S751del)	Gefitinib, 1^st^	PR
	18	G719S				
12	19	Deletion (E746_A750del)		**WT**	Erlotinib,1^st^	PR
13	19	Deletion (E746_A750del)		**WT**	Gefitinib, 1^st^	PR
14	21	L858R	21	L858R	Gefitinib, 1^st^	PD
15	21	L858R	21	L858R	Gefitinib, 2^nd^	PD
16	21	L858R	21	L858R	Gefitinib, 1^st^	PR
17	21	L858R	21	L858R	Gefitinib, 1^st^	PR
18	21	L858R	21	L858R	Gefitinib,1^st^	PR
19	21	L858R	21	L858R	Gefitinib,1^st^	PR
20	21	L858R	21	L858R	Gefitinib,1^st^	PR
21	21	L858R	21	L858R	Gefitinib,1^st^	PR
22	21	L858R	21	L858R	Gefitinib, 2^nd^	PD
			20	**T790M**		
23	21	L858R	21	L858R	None	
	20	S718I	20	**T790M**		
24	21	L858R,	21	L858R	Erlotinib, 1^st^	PR
	20	S784F				
25	21	L858R		**WT**	Gefitinib, 1^st^	PD
26	20	Insertion (D770_N771ins)	20	Insertion (D770_N771ins)	Erlotinib, 3^rd^	PD
27	20	Insertion (V770_D771ins)	20	Insertion(D770_N771ins)	Gefitinib, 1^st^	SD
28	20	Insertion(A763_Y764ins)			None	
	18	K714N	18	K714N		
29	18	Deletion (E709<T910>D)	18	Deletion (E709<T910>D)	Gefitinib, 1^st^	PD
30	19	L747P	19	L747P	Gefitinib, 2^nd^	PD
31	21	R776H,	21	R776H	None	
	20	L861Q				

LN; lymph node, WT; wild type, TKI; tyrosine kinase inhibitor, PR; partial response, SD; stable disease, PD; progressive disease

**Table 3:** Characteristics of 3 cases with discrepancy in PT & LN in major mutations.

Patient #	EGFR mutation in PT	EGFR mutation in LN	Tumor % in PT	Tumor % in LN	Tissue acquisition method for PT	# of aspirations by EBUS-TBNA for LN specimen
12	Deletion 19	WT	> 50%	5%	TTNB	2
13	Deletion 19	WT	> 50%	10%	surgery	2
25	L858R	WT	20	10%	TTNB	3

PT; primary tumor, LN; lymph node, WT; wild type TTNB; transthoracic needle biopsy

Besides the major EGFR mutations, there were other minor EGFR mutation differences between matched PT and LN specimens (Table 2). In one patient with an exon 19 deletion in the matched PT and LN specimens, a G719S mutation was detected in the PT specimen only (case 11). In two cases with an L858R mutation in the matched PT and LN specimens, a T790M mutation was detected in the LN specimen only (cases 22 and 23). Also, in two cases with an L858R mutation in matched PT and LN specimens, rare point mutations in exon 20 (S784F and S781I) were detected in the PT specimens (cases 23 and 24). Among six cases that exhibited minor mutations without major mutations (cases 26–31), the most frequent mutation was an insertion mutation in exon 20 (n = 3, cases 26–28), and we observed differences in these minor mutation patterns between the matched PT and LN specimens in two cases (cases 28 and 31). Altogether, the discordance rate in major or minor EGFR mutations between matched PT and LN specimens was 12% (9/74). Interestingly, among 31 patients with any mutation, additional mutations which were not found in the paired samples, were tended to be detected more frequently in the PTs than the LNs {8/31(case 11, 12, 13, 23, 24, 25, 28 and 31) vs. 2/31(case 22 and 23), p = 0.08).

### Response to EGFR-TKI treatment

Of 74 patients, 42 (56.8%) were treated with EGFR TKIs. Of the 31 patients that had at least one EGFR mutation, 26 were treated with EGFR TKIs (Tables 2 and 4). In the 26 patients with any mutation, the disease control rate was 73.1% (19/26, PR; n = 17, SD; n = 2). Of the 25 patients with major mutations (22 (88%) received TKI treatment, and the disease control rate in this group was 81.8% (18/22, PR; n = 17, SD; n = 1). Sixteen cases with wild type in both PT and LNs were treated with EGFR TKI and the disease control rate in this group was 18.8% (3/16, PR; n = 0, SD; n = 3).

**Table 4:** Results of the TKI treatment according to the EGFR mutations.

	Group1 MT in PT & LN	Group2 MT in PT & WT in LN	Group3 majMT in PT & LN	Group4 majMT in PT & WT in LN	Group5 WT in PT & LN
PR+SD	17/23(73.9%)	2/3(66.7%)	16/19(84.2%)	2/3(66.7%)	3/16 (18.8%)
PD	6/23(26.1%)	1/3(33.3%)	3/19(15.8%)	1/3(33.3%)	13/16(81.2%)

TKI; tyrosine kinase inhibitor, MT; any mutation, majMT; major mutation, WT; wild type, PR; partial response, SD; stable disease, PD; progressive disease

Group 1 vs. Group 2; p = 1.00, Group3 vs. Group4; p = 0.47, Group 1+2 vs. Group5, p = 0.001, Group 3+4 vs. Group5, p = 0.0002

Table 4 shows the results of TKI treatment according to groups of EGFR mutation patterns (Group1; any mutation in PT and any mutation in LN, Group2; any mutation in PT and wild type in LN, Group 3 major mutation in PT and LN, Group4; major mutation in PT and wild type in LN. groups5; wild type in PT and LN). There were no significant differences in response between the concordant mutation group and the discordant mutation group (Group 1 vs. Group 2; p = 1.00, Group3 vs. Group4; p = 0.47). There were significant differences in TKI responses between the mutation group (Group1+2, Group 3+4) and the wild type group (Group 5) (Table 4).

Of the three cases with discrepancies in major mutations between matched PT and LN specimens, the two cases with 19del in the PT specimens-only showed PR, and the one case with L858R in the PT specimen only showed PD. Of the two cases with T790M mutations in the LN specimens-only, one case received gefitinib, and the clinical response was PD. Of the six cases with minor mutations only, four were treated with EGFR TKIs, and the disease control rate of this group was 25% (1/4, SD; n = 1) (Table 2).

## Discussion

In the present study, we observed the discordance rate of major EGFR mutations between LN specimens sampled by EBUS-TBNA and matched PTs in advanced, non-squamous NSCLC in 4.1% (3/74) of cases. The discordance rate major and minor EGFR mutations combined between matched PT and LN specimens was 12% (9/74). This low discordance rate in major mutations shown suggests that EBUS-TBNA-derived LN specimens can be used effectively for the evaluation of EGFR mutation status, and subsequent treatment decisions, in advanced, non-squamous NSCLC. We believe that our results are meaningful because, unlike previous studies that analyzed surgical cases, we analyzed LN samples obtained by EBUS-TNBA from a relatively large number of patients with advanced (stage 3 and 4), non-squamous NSCLC.

The major concern for using LN samples obtained by EBUS-TBNA in EGFR testing is the limitation of a needle technique. For PT specimen, we used biopsy or surgical methods in 57 cases, which may allow larger tumor volumes than LN samples, although we did not compare tumor or DNA volumes between PT and LN. Using EBUS-TBNA, we usually obtain cellblock samples and 21G or 22G needles can retrieve small histologic tissue cores [19, 20]. Lymphocytes mixed with tumors in LN specimen can negatively affect the proportion of tumor cells. However, in the literature, cytologic samples are widely used for molecular analysis [21–23]. Specimens obtained by EBUS-TBNA are also reported to be suitable for EGFR testing [7, 24–28]. In our study, 89% of the LN samples were determined to be feasible for EGFR analysis, which is similar to the results of Navani et al. (90%) and Nakajima et al. (93%)[7, 24]. Number of aspirations by EBUS-TBNA affects the tumor volume. We performed an average of three aspirations by EBUS-TBNA per LN station, as recommended for lung cancer staging [20, 29]. Yarmus et al., a median of four passes with a 21-guage needle is needed to obtain adequate molecular profiling of 95.3%, using EBUS [27]. Although we cannot be certain that we had enough tumor volume with average 3 aspirations, our low discordance rate suggests that LN sampling using EBUS-TBNA is a reasonable approach for tissue acquisition and EGFR testing of NSCLC. Moreover, our discordance rate is similar to, or slightly lower than, the rates published from studies using direct sequencing with surgical specimen (4.5–28.6%)[9, 11–13, 15], which also suggest the efficacy of EBUS-TBNA targeting LNs especially for EGFR testing in advanced NSCLC.

There are other potential explanations for the observed discrepancies in EGFR mutation testing between PT and LN specimens. In this study, we performed direct sequencing, which is regarded as the historical gold standard for EGFR testing[18, 21], however, direct sequencing has a low analytic sensitivity and a high level of false negative results. Generally, to be reliably detected by this method, the percentage of tumor cells should be present in approximately 20–30% of a sample[30–32]. We used microdissection to enrich for tumor cell content, but we did not use an enriched PCR method. In this study, overall, the tumor cell proportion was not different between PT and LN specimens; however, the three cases that showed discrepancies in major EGFR mutations between paired samples had a low proportion of tumor cells (5–10%) in the LN specimens. Considering major and minor EGFR mutations altogether, the discordance rate in between matched PT and LN specimens was 12%. The additional mutations that were not found in the paired samples, were tended to be detected more often in PT than LN specimens, which might also related to the sensitivity issue. A variety of methods, such as peptide nucleic acid (PNA)-clamping, mutant-enriched PCR, and amplification-refractory mutation system (ARMS), have higher sensitivity and have been employed as potential alternatives to direct sequencing[30, 33–38]. Therefore, further studies using these methods are needed to evaluate the validity of using EBUS samples in EGFR mutation testing.

Another potential cause of the observed discrepancies in EGFR mutation testing between PT and LN specimens is tumor heterogeneity. The issue of tumor heterogeneity between PT and LN was not fully evaluated and not yet concluded. Several studies have demonstrated heterogeneity of EGFR mutation status in different ‘primary tumor’ fragments. For example, Bai et al. analyzed EGFR mutation status in various regions of 79 NSCLC tumors and reported that 38% of the tumors showed heterogeneity in EGFR mutations[39]. Taniguchi et al. examined 21 NSCLC patients with EGFR mutations and found that 15 specimens consisted only of cells with EGFR mutations, but the remaining 6 contained both mutated and non-mutated cells[40]. However, according to a study by Yatabe et al., no discrepancies in EGFR mutation status were found in three separate parts of tumor specimen from 50 patients[41]. In terms of EGFR mutation status between PT and LN, Wei et al. performed quantitative measurement of EGFR mutation ratio. They observed that the EGFR mutation ratios detected in different sites of PTs were highly concordant, whereas the EGFR mutation ratios in metastatic LNs were lower than those in PTs, which might suggest that EGFR mutations may not be essential for metastasis[42]. We do not know whether, and how much, the discordance in EGFR mutations between PT and LN specimens is related to intratumoral heterogeneity and the metastasis of specific clones, and more studies are clearly required.

In this study, we found that the response to EGFR-TKI treatment in patients with major EGFR mutations (82%) was similar to those previously published [1, 2]. EGFR mutations in PTs and metastatic LNs are reported to be associated with EGFR-TKI response. Shimizu at al. compared EGFR mutation status in PT and LN surgical specimens from 70 patients, and identified major mutations in PT and LN specimens in 11 patients and in PT specimens-only in 10 patients[14]. Importantly, the disease control rate was tended to be higher in the concordant mutant group than in the PT-only mutant group (p = 0.06). In our study, we could not observe significant differences in response between the concordant mutation group and the discordant mutation group. We observed PD in one and PR in two of three cases with discrepancies in major EGFR mutations. We also observed PD in one case with a T790M mutation, which was associated with EGFR-TKI resistance. It was reported that baseline T790M mutations before treatment generally occur concurrently with another EGFR sensitizing mutation, and are associated with a decreased sensitivity to EGFR TKIs [43, 44]. Although we did not find the association between the discrepant mutation and response to EGFR TKIs statistically, however, our data suggest that mutation testing in different lesions can provide additional useful information for patient management.

This study has several additional limitations. Due to the retrospective sample collection, we did not perform the EBUS-TBNA for the purpose of tissue acquisition and molecular testing. In 40% of the cases (stage 3), EBUS-TBNA was performed for staging. As mentioned above, this could affect sample amount and, subsequently, the results of the EGFR mutation test. Furthermore, due to the nature of the retrospective study, we performed the EGFR testing using FFPE tissue sections, and there were intervals between testing of the PT and LN specimens, which could have also affected the results. However, the adequacy of stored FFPE samples for EGFR mutation testing has been previously reported[45].

## Conclusions

In conclusion, we showed a high concordance rate of major EGFR mutations between LN specimens sampled by EBUS-TBNA and PTs, suggesting that LN samples obtained by EBUS-TBNA from advanced, non-squamous NSCLC patients can be effectively used for EGFR mutation testing. However, we also observed some differences in EGFR mutations between LN and PT samples, including major or minor discordancy, suggesting that mutation testing in different sites could give additional information. These data further suggest that repeated testing with samples obtained from different sites, or sites showing different response, is appropriate, especially when new treatment decisions are needed. Further studies are needed to evaluate differences in response according to the discrepancy in EGFR mutations between lesions. Moreover, studies with more sensitive methods for EGFR mutation testing, and studies with larger amounts of specimens obtained by EBUS-TBNA, are also required.
